# Ergebnisse nach Plattenstabilisierung der Symphysensprengung

**DOI:** 10.1007/s00113-020-00804-8

**Published:** 2020-04-28

**Authors:** Martin C. Jordan, Veronika Jäckle, Sebastian Scheidt, Lars Eden, Fabian Gilbert, Timo M. Heintel, Hendrik Jansen, Rainer H. Meffert

**Affiliations:** 1 Klinik und Poliklinik für Unfall‑, Hand‑, Plastische und Wiederherstellungschirurgie, Universitätsklinikum Würzburg, Oberdürrbacher Straße 8, 97080 Würzburg, Deutschland; 2 Klinik für Orthopädie und Unfallchirurgie, Universitätsklinikum Bonn, Venusberg-Campus 1, 53127 Bonn, Deutschland

**Keywords:** Beckenring, Beckenfraktur, AO, Fixation, Trauma, Pelvic ring, Pelvic fracture, AO, Fixation, Trauma

## Abstract

**Hintergrund:**

Die Symphysensprengung mit entsprechender Diastase kann durch eine Symphysenplatte stabilisiert werden.

**Fragestellung:**

Welche Beckenverletzungen werden mit einer Symphysenplatte stabilisiert und wie ist das Outcome?

**Material und Methoden:**

Retrospektive Auswertung von 64 Patienten über einen Untersuchungszeitraum von 24 Monaten.

**Ergebnisse:**

Es waren 56 Patienten männlich, 8 weiblich und das mittlere Alter betrug 44 Jahre (SD ± 17). Unfälle im Straßenverkehr waren der führende Grund für die Beckenverletzung. Die Verteilung nach AO-Klassifikation zeigte sich wie folgt: 14-mal B1-, 10-mal B2-, 5‑mal B3-, 23-mal C1-, 9‑mal C2- und 3‑mal C3-Verletzungen. Die Verteilung nach Young und Burgess ergab: 9‑mal APC-I-, 18-mal APC-II-, 13-mal APC-III-, 9‑mal LC-I-, 3‑mal LC-II-, 2‑mal LC-III- und 10-mal VS-Verletzungen. Der mittlere Injury Severity Score (ISS) betrug 32 und die mittlere stationäre Verweildauer 29 Tage (pos. Korrelation *p* ≤ 0,001). Im Verlauf war eine radiologische Implantatlockerung bei 52 Patienten nachweisbar. Therapierelevante Komplikationen gab es in 14 Fällen. Hierbei war das Implantatversagen (*n* = 8) der Hauptgrund für eine operative Revision.

**Diskussion:**

Obwohl die radiologische Implantatlockerung häufig beobachtet wird, ist sie nur selten Grund für einen Revisionseingriff. Kommt es hingegen zum vollständigen Implantatversagen, tritt dies meist innerhalb der ersten postoperativen Wochen auf und ist revisionsbedürftig. Eine frühzeitige Abklärung durch Röntgenbildgebung sollte bei Verdacht erfolgen.

## Abstract (en)

**Background:**

Separation of the pubic symphysis with corresponding diastasis can be stabilized by symphyseal plating.

**Objective:**

Which pelvic injuries are stabilized with symphyseal plating and what is their outcome?

**Material and methods:**

A retrospective evaluation of 64 patients who underwent symphyseal plating over a period of 24 months was conducted.

**Results:**

Of the patients 56 were male and 8 female with a mean age of 44 years (SD ± 17 years). The main cause of the pelvic injuries were traffic accidents. The distribution according to the AO fracture classification was 14 × B1, 10 × B2, 5 × B3, 23 × C1, 9 × C2 and 3 × C3 injuries. The distribution according to the Young and Burgess classification showed 9 × APC I, 18 × APC II, 13 × APC III, 9 × LC I, 3 × LC II, 2 × LC III and 10 × VS injuries. The mean Injury Severity Score (ISS) was 32 (SD ± 17) and the mean inpatient stay was 29 days (SD ± 16 days; positive correlation *p* ≤ 0.001). Radiological implant loosening occurred in 52 patients. 14 patients required treatment for severe complications. The leading reason for revision surgery was implant failure (*n* = 8).

**Conclusion:**

These observations suggest that radiological signs of implant loosening are commonly observed but rarely the reason for revision surgery. Complete implant failures, however; occur mainly within the first postoperative weeks and require early revision. A timely clarification by additional X-ray imaging should be carried out if this is suspected.

## Hintergrund und Fragestellung

Als Verletzung des vorderen Beckenrings kann die Symphysensprengung isoliert oder in Kombination mit ligamentären oder knöchernen Läsionen des hinteren Beckenrings auftreten. Die Instabilität der Beckenverletzung ergibt sich aus dem Ausmaß der symphysären Schädigung und der Verletzungsart des hinteren Beckenrings. Bei Verdacht auf eine instabile Beckenverletzung kann präklinisch ein Beckengurt zur temporären Stabilisierung angelegt werden. In der Klinik erfolgt dann die Diagnostik durch konventionelle Röntgenbilder (a.-p., Inlet, Outlet) oder eine Computertomographie [1]. Bei relevanter symphysärer Diastase besteht die Möglichkeit, die bindegewebige Gelenkzerreißung der Symphyse mit einer Symphysenplatte zu stabilisieren [2, 3]. Hierbei handelt es sich nicht um eine klassische Osteosynthese, sondern um eine temporäre Stabilisierung der Faserknorpelverletzung, welche den Kontakt der Schambeinäste bis zur sicheren Heilung der Symphyse sicherstellt [4]. Abhängig vom Verletzungsmuster am hinteren Beckenring ist eine Ergänzung der Symphysenplatte durch unterschiedliche posteriore Stabilisierungsverfahren erforderlich (Plattenosteosynthese, SI-Schrauben, spinoalare Aufhängung etc.).

Folgende Fragen sollen durch diese Studie beantwortet werden: 1) Erhebung des Unfallmechanismus und Klassifikation eines Patientenkollektivs mit Symphysensprengung, 2) Analyse des operativen Vorgehens, 3) Auswertung der radiologischen Verlaufskontrollen und 4) systematische Erfassung aufgetretener Komplikationen.

## Material und Methoden

### Datenerhebung und -aufarbeitung

Es handelt sich um retrospektiv erhobene und irreversibel anonymisierte Daten. Eingeschlossen wurden alle Patienten aus den Jahren 2006–2016, welche aufgrund einer Beckenverletzung in die Notaufnahme eines überregionalen Traumazentrums eingeliefert wurden. Für die Erstellung der Datenbank wurden zuvor definierte Ein- und Ausschlusskriterien festgelegt. Eingeschlossen wurden alle Patienten mit unfallbedingter Symphysensprengung (ICD S33.4), bei denen eine suffiziente Bildgebung (Computertomographie) des Beckens vorlag. Ausgeschlossen wurden alle Patienten, die eine rein knöcherne Verletzung des vorderen Beckenrings aufwiesen, Patientinnen mit einer geburtstraumatischen Symphysensprengung sowie Patienten, die während oder kurz nach der Primärversorgung aufgrund der Verletzungsschwere verstarben. Insgesamt wurden 64 Patienten mit operativ stabilisierter Symphysensprengung identifiziert. Der Nachuntersuchungszeitraum betrug 24 Monate. Die Befundung und Klassifikation der Verletzungstypen erfolgte verblindet durch zwei unabhängige, unfallchirurgische Gutachter analog der aktuellen AO/OTA-Klassifikation sowie der Klassifikation nach Young und Burgess in einem doppeltem Durchlauf [5, 6].

### Operationstechnik

Zugang über einen Pfannenstiel-Schnitt, Darstellung der Rektusscheide, Längsinzision der Linea alba, partielle Ablösung der Rektusmuskulatur (wenn nicht bereits abgerissen), retrosymphysäre Präparation unter Schonung der Harnblase und Reposition unter Bildwandlerkontrolle mit entsprechenden Repositionszangen. Zur symphysären Stabilisierung wurden 4‑Loch- oder 6‑Loch-Repositionsplatten aus Stahl verwendet (SPS Matta Pelvic System, Fa. Stryker Kalamazoo, Michigan, USA *n* = 34 oder 3.5 Pelvic Implant, Fa. DePuy-Synthes, Warsaw, Indiana, USA *n* = 30). Je nach Verletzung und Versorgungsart erhielten die Patienten postoperativ eine Röntgen- oder CT-Kontrolle. Eine weitere Röntgenkontrolle wurde nach 6 Wochen durchgeführt. Die Patienten stellten sich innerhalb der ersten 2 Jahre in regelmäßigen Abständen in der Ambulanz vor.

### Gemessene Parameter

Die Häufigkeitsverteilung der Frakturtypen erfolgte nummerisch und prozentual. Die Einteilung des Verletzungsmechanismus wurde anhand des Notarztprotokolls in 5 Kategorien (Motorrad, Sturz, Pkw, Passant/Rad/Pferdesturz, Überrolltrauma) vorgenommen. Das Patientenalter, die stationäre Verweildauer und der Injury Severity Score (ISS) wurden als quantitative Daten erhoben. Die symphysäre Diastase wurde präoperativ in den axialen CT-Schichten an der posterioren Begrenzung der Symphyse gemessen (MERLIN Diagnostic Workcenter von Phönix-PACS). Hierbei wurde unterschieden, ob die Bildgebung mit oder ohne Beckengurt erfolgte. Ob eine interventionelle Gefäßembolisation erforderlich war, wurde ebenfalls dokumentiert. Die Art der operativen Primär- und Sekundärversorgung wurde aufgearbeitet. Anschließend folgte die Auswertung der vorhandenen Bildgebung mit Fokus auf Schraubenlockerung oder Implantatversagen. Als Komplikation wurden therapierelevantes Implantatversagen, Infektionen, Serom/Hämatom oder störende heterotope Ossifikationen gewertet. Außerdem wurden Patienten mit Entfernung der Symphysenplatte im Untersuchungszeitraum erfasst.

### Datenanalyse und Statistik

Eine statistische Auswertung der Verletzungsart, des Verletzungsmechanismus, der Verletzungsschwere sowie aufgetretener Komplikationen wurde durch Analyse der Häufigkeitsverteilungen, Mittelwertvergleiche, Korrelationsbestimmungen und eine Überlebenszeitanalyse durchgeführt (IBM, SPSS 25, Armank, New York, USA). Die Daten wurden in entsprechende Skalenniveaus unterteilt. Für die metrischen Daten wurde eine deskriptive Statistik mit Darstellung des Mittelwerts, Standardabweichung, Minimum und Maximum erstellt. Für die nominalen Daten wurde eine Häufigkeitsverteilung angezeigt. Auf Normalverteilung wurde geprüft. Das Alter (Jahre), die Symphysenweite (mm), die Dauer des stationären Aufenthalts (Tage) und der ISS waren nicht normalverteilt. Die bivariable Korrelationsbestimmung erfolgte unter Berücksichtigung des Skalenniveaus mit entsprechenden Tests. Für die Darstellung der komplikationsfreien Nachuntersuchungszeit nach der Kaplan-Prozedur wurden die Zeit bis zum Auftreten einer Komplikation, das definitive Auftreten einer Komplikation und die AO-Klassifikation als Faktoren gewählt. Nach Erstellung der Tabelle wurden Mittelwert und Median für die komplikationsfreie Nachuntersuchungszeit angegeben. Zur Analyse der komplikationsfreien Nachuntersuchungszeit wurde der Log-Rank-Test verwendet.

## Ergebnisse

### Alter

Die Patienten waren im Mittel 44 Jahre (±17 Jahre; Range: 17 bis 80 Jahre) alt, und die Geschlechterverteilung betrug männlich: weiblich 56 (87,5 %): 8 (12,5 %). Es besteht keine Abhängigkeit zwischen männlichem Geschlecht und erhöhtem ISS (Phi: 0,674; *p* = 0,142).

### Unfallmechanismus und ISS

**Figure 1:**
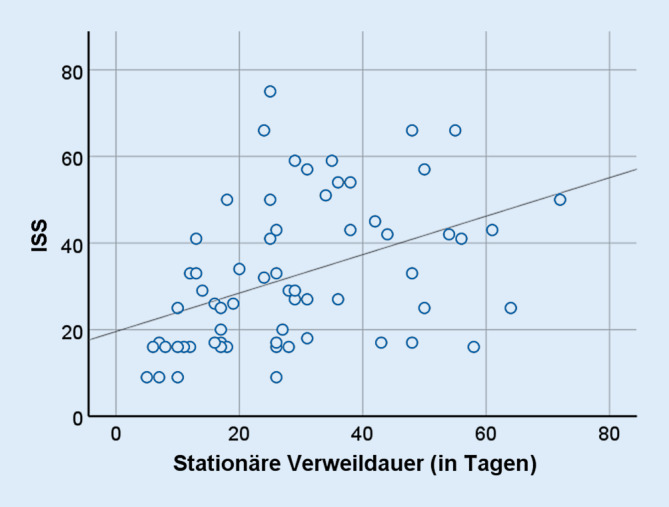

Als Unfallursachen wurden bei 30 (46,9 %) Patienten ein Motorradunfall, bei 17 (26,6 %) ein Sturz, bei 9 (14,1 %) ein Pkw-Unfall, bei 4 (6,3 %) ein Unfall als Passant oder Fahrradfahrer und bei weiteren 4 (6,3 %) ein Überrolltrauma angegeben (Tab. 1). Die Diastase der Schambeinäste bei nichtangelegtem Beckengurt, gemessen im CT, betrug im Mittel 25 mm (±14 mm, *n* = 57), wohingegen die Symphyse bei angelegtem Beckengurt nur 6 mm (±3 mm, *n* = 7) auseinanderwich. Unter Berücksichtigung der Begleitverletzungen betrug der errechnete ISS-Mittelwert 32 (±17). Nur 4 (6 %) Patienten hatten einen ISS unter 16. Die mittlere stationäre Verweildauer belief sich auf 29 Tage (±16). Zwischen dem ISS und der stationären Verweildauer zeigte sich eine signifikante Korrelation (Spearman rho: 0,493; *p* ≤ 0,001; Abb. 1). Außerdem gab es eine signifikante Korrelation zwischen der Diastase der Symphyse in Millimetern (mm) und der Dauer des stationären Aufenthalts (Korrelationskoeffizient nach Pearson 0,39; *p* = 0,003). Eine Abhängigkeit zwischen dem Alter und der Dauer des stationären Aufenthalts bestand nicht. Eine interventionelle Embolisation zur Behandlung arterieller Blutungen im Beckenbereich wurde bei 7 (10,9 %) Patienten innerhalb der ersten 24 h durchgeführt.

**Table 1:** 

Unfallmechanismus	Anzahl der Patienten (*n* = 64)
Motorrad	30
Sturz	17
Pkw	9
Überrolltrauma	4
Passant/Rad/Pferd	4

### Klassifikation

**Figure 2:**
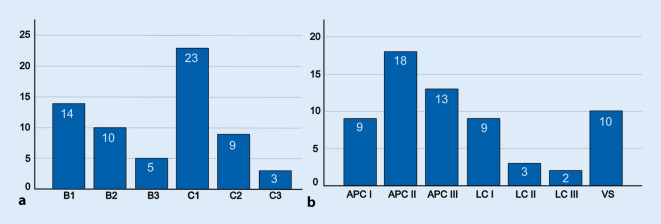

Die Verteilung der Beckenverletzungen nach AO-Klassifikation zeigte sich wie folgt: 14 (21,9 %) B1, 10 (15,6 %) B2, 5 (7,8 %) B3, 23 (35,9 %) C1, 9 (14,1 %) C2 und 3 (4,7 %) C3. Die Klassifikation nach Young und Burgess ergab folgende Verteilung: 9 (14,1 %) APC I, 18 (28,1 %), APC II, 13 (20,3 %) APC III, 9 (14,1 %) LC I, 3 (4,7 %) LC II, 2 (3,1 %) LC III und 10 (15,6 %) VS (Abb. 2). Offene Frakturen gab es bei 4 (6,3 %) Patienten.

### Operatives Vorgehen

Entsprechend dem primären Einschlusskriterium umfasste die operative Versorgung bei allen Patienten die Anwendung einer Symphysenplatte (4-Loch *n* = 60; 6‑Loch *n* = 4). Jedoch wurden 21 (32,8 %) Patienten initial mit einem supraacetabulären Fixateur externe stabilisiert und erst im Verlauf mit einer Symphysenplatte versorgt. Bei 33 (51,6 %) Patienten erforderte die Frakturmorphologie eine zusätzliche Stabilisierung des hinteren Beckenrings. Hierbei wurde zwischen transiliosakraler Schraube (*n* = 24), dorsaler Plattenosteosynthese (*n* = 6), spinoalarer Aufhängung (*n* = 2) und dorsaler Gewindestange (*n* = 1) unterschieden. Die perkutane transiliosakrale Schraubentechnik zur dorsalen Stabilisierung setzte sich im zeitlichen Verlauf vermehrt durch.

### Radiologische Implantatlockerung und Implantatentfernung

**Figure 3:**
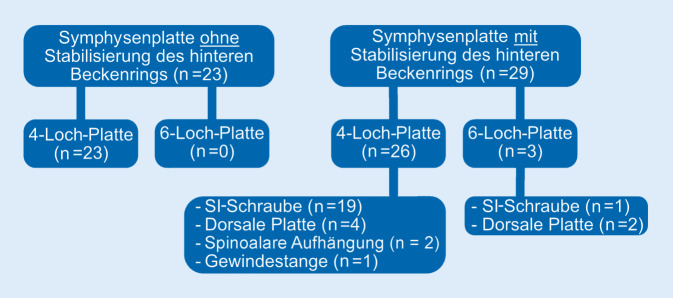

In den radiologischen Verlaufskontrollen konnte bei 52 (81,3 %) Patienten eine Schraubenlockerung nachgewiesen werden (Abb. 3). Bei 15 (23,4 %) Patienten erfolgte eine Implantatentfernung in unserer Klinik. Dabei korrelierte das Patientenalter schwach mit der Implantatentfernung: Je jünger ein Patient war, desto häufiger wurde eine Entfernung vorgenommen (Pearson 0,475; *p* ≤ 0,001).

### Komplikationen

**Figure 4:**
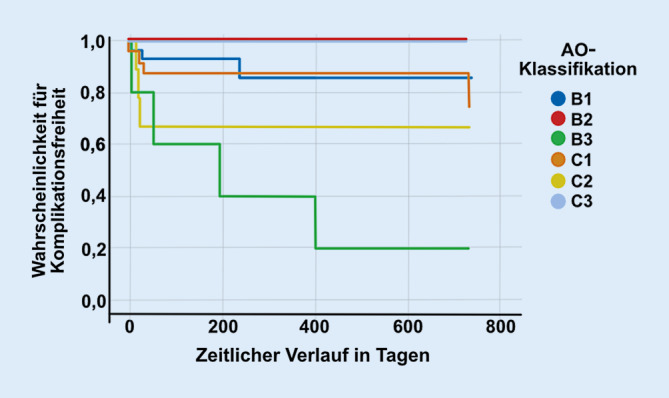

Relevante Komplikationen gab es in 14 (21,9 %) Fällen, von denen alle operativ revidiert werden mussten. Bei 8 Patienten trat ein therapierelevantes Implantatversagen auf, bei 3 eine Infektion, 2 weitere entwickelten ein Hämatom/Serom, und bei einem Patienten entwickelte sich eine störende heterotope Ossifikation (Tab. 2). Von diesen 14 Patienten mit einer Komplikation hatten 4 eine isolierte Symphysensprengung, welche mit einer Symphysenplatte stabilisiert wurde und 10 eine kombinierte Verletzung des vorderen und hinteren Beckenrings, welche beide eine operative Stabilisierung erforderten. Es gab keine Korrelation zwischen isolierter Läsion des vorderen bzw. Läsion des vorderen und hinteren Beckenrings und dem Risiko, eine Komplikation zu erleiden (Chi-Quadrat: 2,832; *p* = 0,092). Außerdem gab es keine Korrelation zwischen dem Auftreten einer Komplikation und der Implantatwahl (Chi-Quadrat: 2,181; *p* = 0,140) (Abb. 4). Die komplikationsfreie Zeit der jeweiligen Verletzungstypen nach der AO-Klassifikation wurde durch den Log-Rank-Test (Mantel-Cox) ausgewertet und ergab einen Unterschied zwischen den Frakturtypen (Chi-Quadrat: 19,207; *p* = 0,02). Die Komplikationen bei B3-Verletzungen sind durch eine inhomogene Verteilung (Lockerung, Infektion, heterotope Ossifikation) gekennzeichnet, weshalb hier eher von einer akzidentiellen Kumulation als von einer tatsächlichen Tendenz ausgegangen werden kann.

**Table 2:** 

Art der Komplikation	Anzahl der Patienten (*n* = 14)
Implantatversagen	8
Infektion	3
Hämatom/Serom	2
Heterotope Ossifikation	1

## Diskussion

### Unfallmechanismus und Verletzungstypen

Dalal et al. berichteten 1989 von ihrem Patientenkollektiv, indem 57,4 % der Beckenverletzungen durch Pkw-Unfälle und nur 9,3 % durch Motorradunfälle verursacht wurden [7]. Auch bei Pereira et al. sind die PKW-Unfälle im Jahr 2017 führend [8]. Auch wenn sich die Daten aufgrund der regionalen und zeitlichen Unterschiede nur eingeschränkt vergleichen lassen, so ist doch ein Unterschied zu unseren Daten mit deutlich mehr Motorradfahrern vorhanden. Motoradfahren prädestiniert durch die anterior-posteriore Krafteinwirkung im Falle eines Unfalls besonders für das Auftreten einer Symphysensprengung und erfreut sich als Freizeitbeschäftigung möglicherweise zunehmender Beliebtheit oder aber die verbesserte Fahrzeugsicherheit der vergangenen Jahre bewirkt einen Rückgang betroffener Pkw-Fahrer. Absolute Zahlen liegen zu dieser Frage aber nicht vor. Dass insbesondere Druck- (AP) und Scherkraft (VS) die Zerreißung der Symphyse in unserer Population bedingen, deckt sich mit der vorhandenen Literatur [5, 9, 10]. Als neue Erkenntnis ist die positive Korrelation zwischen ISS und stationärem Aufenthalt zu nennen, was als Referenz bei der Rehabilitationsplanung verwendet werden kann.

### Operative Versorgung

Die korrekte Klassifizierung der Beckenringverletzung kann gelegentlich Probleme bereiten. Eine nachweisbare Inter- und Intraobserver-Variabilität ist einer der Gründe hierfür [11]. Eine weitere Schwierigkeit der präoperativen Planung kann die CT-Bildgebung mit angelegtem Beckengurt sein, da hierdurch die Instabilität der Symphyse möglicherweise unterschätzt wird. Der hier vorgestellte Unterschied zwischen geschlossenem und offenem Beckengurt in der CT-Diagnostik ist eindrücklich. Auch Swartz et al. thematisierten bereits das Problem der Fehlinterpretation von Beckenverletzungen durch angelegten Beckengurt [12]. Um die Maskierung einer Symphysenverletzung zu vermeiden, kann bei kreislaufstabilen Patienten die Bildgebung (im Konsens mit anderen beteiligten Disziplinen) auch ohne Beckengurt erfolgen. Es wird aber darauf hingewiesen, dass dieser Punkt Gegenstand kontroverser Diskussionen ist und die Etablierung eines einheitlichen „Clear-the-pelvis“-Algorithmus noch aussteht [1]. Gibt es in der klinischen Untersuchung oder der CT-Diagnostik weiterhin Unklarheit bezüglich der Stabilität, so ist die manuelle Stabilitätstestung unter Durchleuchtung der konsekutive Schritt zur weiteren Beurteilung mit hoher Sensitivität [12]. Die MRT-Diagnostik spielt in diesem Kontext nur eine untergeordnete Rolle [13].

Ein weiterer Faktor ist die adäquate Stabilisierung des hinteren Beckenrings. Biomechanische Studien zeigen, dass nach isolierter anteriorer Stabilisierung eine deutliche Restbewegung im geschädigten Iliosakralgelenk besteht [14]. Klinische Studien belegen eine deutlich geringere Lockerungsrate bzw. Rediastase der Schambeinäste bei kombinierter Verwendung einer Symphysenplatte und SI-Schraube [15]. Ab wann eine Versorgung des hinteren Beckenrings bei unvollständiger Läsion aber durchgeführt werden sollte, ist weiterhin unklar. Metz et al. simulierten APC-II-Verletzungen in einer Kadaverstudie und konnten keine Unterschiede zwischen der Gruppe mit Symphysenplatte und der Gruppe mit Symphysenplatte und SI-Schraube nachweisen. Da insgesamt nur 7 Becken getestet wurden, sind sicherlich noch weitere Studien erforderlich, um diese Feststellung abschließend zu verifizieren [16]. Eine Umfrage aus dem Jahr 2017 zur Versorgung von APC-II-Verletzungen ergab, dass die Verwendung einer Symphysenplatte und einer SI-Schraube die gegenwärtig populärste Technik im Vereinigten Königreich ist [17]. Es liegt letztlich beim Operateur, die Instabilität des hinteren Beckenrings adäquat zu beurteilen.

Auch der Einfluss des Implantatdesigns wird diskutiert. Moed et al. zeigten keinen Vorteil der winkelstabilen gegenüber der nichtwinkelstabilen Symphysenplatte [18]. Vier- oder 6‑Loch-Platten sollten verwendet werden, da 2‑Loch-Platten aufgrund der Rotationsinstabilität zu einer anterior-posterioren Dislokation der Schambeinäste neigen [19]. In dem vorgestellten Patientenkollektiv wurde nur bei 4 Patienten eine 6‑Loch-Platte verwendet. Ein Unterschied zwischen den Platten konnte nicht nachgewiesen werden. Weiterhin sind die Kompressionskräfte eine Plattenosteosynthese mit dynamischer Kompression vorteilhaft, da sie eine straffe Vernarbung im Bereich der Symphyse begünstigen [20]. Auch wenn die Symphysenplatte im Vergleich zum Fixateur eine höhere Stabilität durch die frakturnähere Fixation aufweist [21, 22], so können individuelle Risikofaktoren (Infektion, Adipositas, Demenz, M. Parkinson etc.) dennoch die Behandlung im Fixateur externe erfordern, wobei auch hier Probleme wie Lockerung und „Pin-tract“-Infektionen bestehen. Inwieweit der Fixateur interne eine Rolle spielt, muss noch geklärt werden [23, 24]. Abschließend sei noch erwähnt, dass bei einer der hier nachuntersuchten Platten die Verwendung von 3,5-mm- als auch 4,5-mm-Schrauben regulär möglich ist. Auch wenn unsere Daten diesen technischen Aspekt nicht eindeutig beleuchten, so könnte der größere Schraubendurchmesser möglicherweise ein Abbrechen der Schrauben verhindern oder eine Schraubenlockerung verzögern.

### Radiologische Implantatlockerung

Bei den Komplikationen muss zwischen der subklinischen Implantatlockerung ohne größere Diastase der Schambeinäste und dem Auftreten einer klinisch revisionsbedürftigen Komplikation, wie z. B. einem vollständigen Ausriss der Schrauben, unterschieden werden. Ersteres kann bei einem Großteil der Patienten im Verlauf beobachtet werden. Die radiologisch nachweisbare Lockerungsrate in dieser Arbeit deckt sich mit der Literatur: Colling et al. beobachteten eine subklinische Schraubenlockerung in 78 % der Fälle. In dieser Studie konnte in den a.-p.-Röntgenbildern im Verlauf auch eine Aufweitung der Symphyse bei liegender Platte von 4,9 mm auf 8,4 mm beobachtet werden, ohne dass hieraus ein klinischer Nachteil oder eine Operationsindikation entstand. Erst ab einer Veränderung der Diastase über 10 mm stieg das Risiko für ein relevantes Implantatversagen [25]. Die Verteilung der Implantatlockerung in Bezug auf die Versorgungsstrategie zeigt Abb. 3.

### Komplikationen

**Figure 5:**
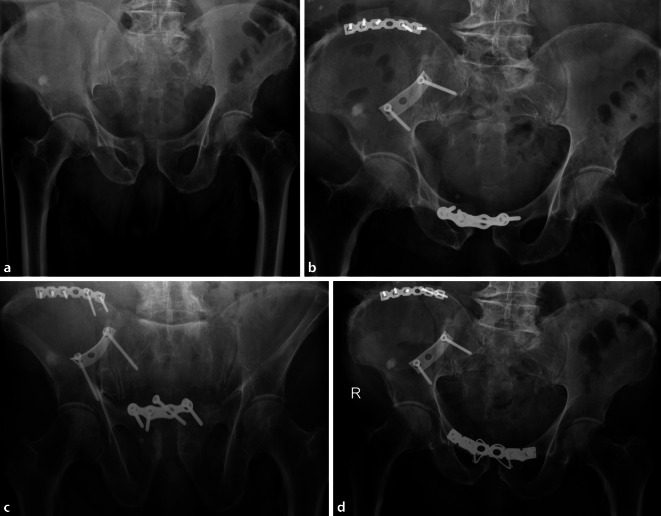

**Figure 6:**
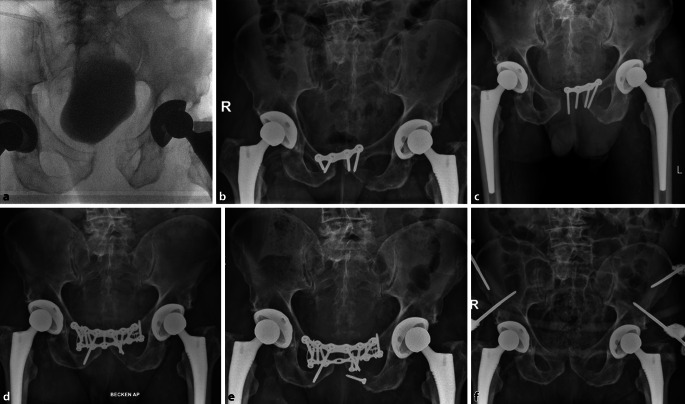

Die relevanten Komplikationen umfassen Implantatversagen, Infektionen, Hämatome oder auch heterotope Ossifikationen. Ein Implantatversagen bei einer 79 Jahren alte Patientin mit instabiler Beckenringverletzung (AO:61-C1.1d, LC II) nach Sturz zeigt Abb. 5a). Es folgte die operative Stabilisierung des vorderen und hinteren Beckenrings (Abb. 5b). Bereits nach 9 Tagen kam es zur Lockerung der Symphysenplatte (Abb. 5c), woraufhin der vordere Beckenring mit einer Kombination aus längerer Platte und Cerclage dauerhaft stabilisiert werden konnte (Abb. 5d**)**. Einen 80 Jahre alten Patienten mit Beckenringverletzung (AO:61-B2.3d, APC II) und Versorgung mit Symphysenplatte zeigt Abb. 6a,b. Drei Wochen postoperativ kam es zum Ausriss des Implantats (Abb. 6c) und zur Revision mit Doppelplattenosteosynthese sowie Cerclage (Abb. 6d). Aufgrund eines Infekts erneute Lockerung und Auseinanderweichen der Schambeinäste. Die Kontrolle des Infekts gelang nur durch vollständige ME, serielles Débridement, lokale/systemische Antibiotika und Fixateur externe (Abb. 6f). Vergleicht man die hier vorgestellte Komplikationsrate von > 20 % mit bisherigen Studien, so ist diese durchaus als hoch einzustufen. Morris et al. berichten 2012 in ihrem Patientenkollektiv von 43 % Implantatversagen, 4 % Repositionsverlust, 3 % Revision der Osteosynthese und 2 % Infektionen [9]. Ptunis et al. zeigten in ihrer Fallserie ein Implantatversagen von 31 %, 12 % Repositionsverlust und 8 % Revisionsrate der anterioren Fixation [26]. Auch Eastman et al. veröffentlichten im Jahr 2016 eine retrospektive Analyse von 126 Patienten mit einer Symphysensprengung. 11 % der Patienten hatten postoperativ ein Implantatversagen, wobei viele dieses Implantatversagen als hörbares Knacken bemerkten [10]. Die tatsächliche Prozentzahl der Komplikationen variiert zwischen den einzelnen Studien und ist von der genauen Definition einer für die Autoren relevanten Komplikation abhängig. In den genannten Studien fand das Implantatversagen ebenfalls in den ersten Wochen nach der Operation statt, weshalb bei Verdacht frühzeitig eine Röntgendiagnostik durchgeführt werden sollte. Zugangsbedingte Komplikationen wie Hämatome oder Wundinfektionen könnten in Zukunft vielleicht durch minimal-invasive Zugänge zur Symphyse reduziert werden [27].

Das retrospektive, monozentrische Design unserer Studie birgt das Risiko der Fehlinterpretation. Ein Bias ist auch durch die isolierte Betrachtung von Beckenverletzungen mit operativ behandelter Symphysensprengung denkbar und muss berücksichtigt werden [8, 28]. Eine prospektive, multizentrische Studie könnte in Bezug auf die kombinierte Versorgung von Symphysenplatte und SI-Schraube bei der klassischen AP-Verletzung hilfreich für zukünftige Behandlungsempfehlungen sein.

Zusammenfassend zeigt das vorgestellte Patientenkollektiv eine hohe Komplikationsrate, welche sich aber bei genauerer Analyse und Vergleich mit der vorhandenen Literatur relativiert. Da die Implantatlockerung der führende Komplikationsgrund ist, sollten bei der Operation alle Möglichkeiten zur Vermeidung einer frühzeitigen Lockerung genutzt werden. Hierbei sind insbesondere die Schraubenplatzierung und Schraubenlänge zu nennen. Auf Grundlage eines CT-Simulationsmodells empfehlen Michelitsch et al. bei idealer Schraubenplatzierung eine mittlere Schraubenlänge von 65–68 mm für die medialen und 44–45 mm für die lateralen Schrauben [29]. Mit entsprechender Inklination in der koronaren Ebene können sich mediale und laterale Schraube überkreuzen, was ebenfalls die biomechanische Ausreißkraft erhöht [30]. Der knöcherne Korridor reicht für 3,5-mm- als auch für 4,5 mm-Schrauben [31]. Eine Stabilisierung des hinteren Beckenrings sollte bei entsprechender Schädigung durchgeführt werden.

## Fazit für die Praxis

- Die Symphysensprengung entsteht überwiegend durch Unfälle im Straßenverkehr. Der führende Traumamechanismus ist eine anterior-posteriore Kompression, welche besonders häufig bei Motorradfahrern auftritt.
- Es zeigt sich eine deutlich positive Korrelation zwischen Verletzungsschwere und stationärer Verweildauer.
- Die CT-Diagnostik mit angelegtem Beckengurt kann das reale Ausmaß der Beckenschädigung maskieren, da die Diastase der Symphyse durch den komprimierenden Beckengurt geringer erscheint, als es möglichweise der Fall ist.
- Die radiologische Schraubenlockerung ohne zunehmende Diastase ist meist als unwesentlicher Nebenbefund zu werten.
- Kommt es aber zu einem vollständigen Implantatversagen, tritt dies vermehrt innerhalb der ersten Wochen nach der Initialversorgung auf, weshalb entsprechende Vigilanz und rechtzeitige Diagnostik entscheidend sind.

## References

[1] Schweigkofler U, Wohlrath B, Paffrath T, Flohé S, Wincheringer D, Hoffmann R, Trentzsch H (2016). „Clear-the-Pelvis-Algorithmus“ Handlungsempfehlung zur Freigabe des Beckens nach nicht invasiver Stabilisierung mittels Beckengurt im Rahmen der Schockraumversorgung. Z Orthop Unfall.

[2] Becker I, Woodley SJ, Stringer MD (2010). The adult human pubic symphysis: a systematic review. J Anat.

[3] Pohlemann T, Gänsslen A (1999). Die Operation der Symphysensprengung. Orthop Traumatol.

[4] Icke C, Koebke J (2014). Normal stress pattern of the pubic symphysis. Anat Cell Biol.

[5] Young JW, Burgess AR, Brumback RJ, Poka A (1986). Pelvic fractures: value of plain radiography in early assessment and management. Radiology.

[6] Meinberg EG, Agel J, Roberts CS, Karam MD, Kellam JF (2018). Fracture and dislocation classification compendium-2018. J Orthop Trauma.

[7] Dalal SA, Burgess AR, Siegel JH (1989). Pelvic fracture in multiple trauma: classification by mechanism is key to pattern of organ injury, resuscitative requirements, and outcome. J Trauma.

[8] Pereira GJC, Damasceno ER, Dinhane DI, Bueno FM, Leite JBR, Ancheschi BDC (2017). Epidemiology of pelvic ring fractures and injuries. Rev Bras Ortop.

[9] Morris SA, Loveridge J, Smart DK, Ward AJ, Chesser TJ (2012). Is fixation failure after plate fixation of the symphysis pubis clinically important?. Clin Orthop Relat Res.

[10] Eastman JG, Krieg JC, Routt ML (2016). Early failure of symphysis pubis plating. Injury.

[11] Berger-Groch J, Thiesen DM, Grossterlinden LG, Schaewel J, Fensky F, Hartel MJ (2019). The intra- and interobserver reliability of the Tile AO, the Young and Burgess, and FFP classifications in pelvic trauma. Arch Orthop Trauma Surg.

[12] Swartz J, Vaidya R, Hudson I, Oliphant B, Tonnos F (2016). Effect of pelvic binder placement on OTA classification of pelvic ring injuries using computed tomography. Does it mask the injury?. J Orthop Trauma.

[13] Nuchtern JV, Hartel MJ, Henes FO (2015). Significance of clinical examination, CT and MRI scan in the diagnosis of posterior pelvic ring fractures. Injury.

[14] Stuby FM, Lenz M, Doebele S (2017). Symphyseal fixation in open book injuries cannot fully compensate anterior SI joint injury—a biomechanical study in a two-leg alternating load model. PLoS ONE.

[15] Avilucea FR, Whiting PS, Mir H (2016). Posterior fixation of APC-2 pelvic ring injuries decreases rates of anterior plate failure and malunion. J Bone Joint Surg Am.

[16] Metz RM, Bledsoe JG, Moed BR (2018). Does posterior fixation of partially unstable open-book pelvic ring injuries decrease symphyseal plate failure? A biomechanical study. J Orthop Trauma.

[17] Gill JR, Murphy C, Quansah B, Carrothers A (2017). Management of the open book APC II pelvis: survey results from pelvic and acetabular surgeons in the United Kingdom. J Orthop.

[18] Moed BR, O’Boynick CP, Bledsoe JG (2014). Locked versus standard unlocked plating of the symphysis pubis in a type-C pelvic injury: a cadaver biomechanical study. Injury.

[19] Sagi HC, Papp S (2008). Comparative radiographic and clinical outcome of two-hole and multi-hole symphyseal plating. J Orthop Trauma.

[20] Pizanis A, Garcia P, Santelmann M, Culemann U, Pohlemann T (2013). Reduction and fixation capabilities of different plate designs for pubic symphysis disruption: a biomechanical comparison. Injury.

[21] Vigdorchik JM, Esquivel AO, Jin X, Yang KH, Onwudiwe NA, Vaidya R (2012). Biomechanical stability of a supra-acetabular pedicle screw internal fixation device (INFIX) vs external fixation and plates for vertically unstable pelvic fractures. J Orthop Surg Res.

[22] Cavusoglu AT, Erbay FK, Ozsoy MH, Demir T (2017). Biomechanical comparison of supraacetabular external fixation and anterior pelvic bridge plating. Proc Inst Mech Eng H.

[23] Jordan MC, Brems AC, Heintel T, Jansen H, Hoelscher-Doht S, Meffert RH (2019). The anterior subcutaneous pelvic ring fixator: no biomechanical advantages compared with external fixation. J Bone Joint Surg Am.

[24] Vaidya R, Martin AJ, Roth M, Nasr K, Gheraibeh P, Tonnos F (2017). INFIX versus plating for pelvic fractures with disruption of the symphysis pubis. Int Orthop.

[25] Collinge C, Archdeacon MT, Dulaney-Cripe E, Moed BR (2012). Radiographic changes of implant failure after plating for pubic symphysis diastasis: an underappreciated reality?. Clin Orthop Relat Res.

[26] Putnis SE, Pearce R, Wali UJ, Bircher MD, Rickman MS (2011). Open reduction and internal fixation of a traumatic diastasis of the pubic symphysis: one-year radiological and functional outcomes. J Bone Joint Surg Br.

[27] Kuper MA, Trulson A, Trulson IM (2019). EASY (endoscopic approach to the symphysis): a new minimally invasive approach for the plate osteosynthesis of the symphysis and the anterior pelvic ring-a cadaver study and first clinical results. Eur J Trauma Emerg Surg.

[28] Court-Brown CM, Caesar B (2006). Epidemiology of adult fractures: a review. Injury.

[29] Michelitsch C, Nguyen-Kim TD, Jentzsch T, Simmen HP, Werner CM (2016). Computed tomography-based three-dimensional visualisation of bone corridors and trajectories for screws in open reduction and internal fixation of symphysis diastasis: a retrospective radiological study. Arch Orthop Trauma Surg.

[30] Robert KQ, Chandler R, Baratta RV, Thomas KA, Harris MB (2003). The effect of divergent screw placement on the initial strength of plate-to-bone fixation. J Trauma.

[31] Link BC, Ha NB, Solomon LB, Rickman M (2017). Defining the pubic symphysis angle with respect to the coronal plane—clinical and biomechanical considerations. Injury.

